# The Equality Health-CVD intervention: protocol for a feasibility study of a home-based multi-component cardiovascular support intervention

**DOI:** 10.1186/s40814-026-01819-5

**Published:** 2026-04-08

**Authors:** Anna Pennington, Kelly Morgan, Jonathan Hewitt, Tracy Daszkiewicz

**Affiliations:** 1 Gwent Public Health Team, Aneurin Bevan University Health Board, Caerleon, UK; 2 School of Social Sciences, Cardiff University, Cardiff, UK; 3 Centre for Development, Evaluation, Complexity and Implementation in Public Health Improvement (DECIPHer,) School of Social Sciences, Cardiff University, Cardiff, UK; 4 School of Medicine , Cardiff University, Cardiff, UK; 5 Aneurin Bevan University Health Board, Ysbyty Ystrad Fawr, Ystrad Mynach, UK; 6 Stroke, HSCRW, Cardiff, UK

## Abstract

**Background:**

Cardiovascular diseases comprise a range of conditions affecting the heart and blood vessels, with numerous underlying modifiable determinants that raise the risk of heart disease, stroke, and peripheral vascular disease. Many of these risk factors could be mitigated through community-based health improvement interventions, which include physiological assessments, health behaviour coaching, psychosocial factors, and wider determinants of health support. Despite their potential, such interventions are seldom integrated into standard secondary care provision. This feasibility study aims to assess the feasibility and acceptability of the equality health-cardiovascular disease (Equality Health-CVD) intervention, a 12-week, place-based intervention for patients with vascular disease.

**Methods:**

The study will use a single-arm 12-week pilot study based in Wales, UK. A total of 20 participants will be recruited. Quantitative data (questionnaire and physical health assessments) will be collected at week 1 (baseline) and week 12. Questionnaires will include PROMIS 10, South Wales Social Wellbeing Scale and EuroQol-5D tools. Health assessments will collect data on body mass index, waist circumference, heart rate and blood pressure. One-to-one interviews will be conducted with a sub-sample of participants (*n* = 10–15) and intervention deliverers (*n* = 2). Data on participant recruitment and retention and the fidelity of intervention implementation will also be collected.

**Discussion:**

This study will provide important findings on the feasibility and acceptability of the Equality Health-CVD. Findings are critical for guiding future directions on intervention implementation and efficacy.

**Trial registration:**

ISRCTN11889657.

## Introduction and background

Cardiovascular disease (CVD), stroke and peripheral vascular disease (PVD) represent leading causes of mortality and morbidity globally, contributing to an estimated annual societal expenditure of £60 billion in the United Kingdom alone [1–5]. While these conditions impact people of all socioeconomic backgrounds, health inequalities are particularly pronounced among populations experiencing deprivation, with poorer outcomes driven by social determinants of health (SDoH) [6–8].

Stroke, transient ischemic attack (TIA), and PVD share common modifiable risk factors including atrial fibrillation, hypertension, dyslipidaemia, smoking, and diabetes [9, 10]. PVD is the third most common cause of atherosclerotic morbidity following CVD and stroke and further complicates the burden of vascular conditions [11], with patients diagnosed with PVD facing significantly greater risk of experiencing more severe cardiovascular events [12].

Preventative strategies, targeting lifestyle changes and risk factor management, can reduce recurrent vascular events; however, their success is often constrained by socioeconomic barriers [13–17].

As Professor Sir Michael Marmot articulates: “Why treat people and send them back to the conditions that made them sick?” p4 [18], emphasising that clinical treatment without addressing underlying social determinants perpetuates health inequalities. Despite progress in health policy recognising the influence of social gradient and social determinants of health (SDoH) on health outcomes [19, 20], achieving equitable improvements across all social gradients remains slow [21, 22].

### The evidence gap

The burden of CVD, stroke and PVD on patients, caregivers, the health system, and the broader economy is substantial, leading to reduced quality of life, high rates of hospitalisations, increased healthcare costs, and premature mortality [23, 24]. While the National Health Service (NHS) implements interventions promoting lifestyle modifications and risk management [25], there is a notable gap in research focusing on interventions that effectively address adverse SDoH, such as food and housing insecurity, violence, and poverty [26–28]. Although research has begun to explore the NHS's efforts to address inequalities [29, 30] and evidence demonstrates benefits of addressing social determinants of health [31, 32], comprehensive research on interventions that bring about significant change particularly in secondary care using place-based approaches remains limited.

Critically, no existing secondary care intervention integrates all four dimensions recommended by the adapted Labonte model [33]: (1) physiological impacts (health monitoring), (2) health behaviours (coaching and goal setting using what matters conversation), (3) psychosocial factors, (social support and connection), and (4) wider determinants of health (practical support addressing structural barriers). The National Institute for Health and Care Excellence [34] specifically recommends adopting place-based approaches integrating these dimensions to reduce health inequalities. National public bodies emphasise collaborative strategies to improve population health [35], and the NHS is uniquely positioned to address SDoH, a role intently endorsed across the UK’s four devolved nations [35, 36].

### Study rationale and novelty

This feasibility study addresses this evidence gap by testing the Equality Health-CVD intervention, a novel 12-week, home-based intervention operationalising all four Labonte model dimensions for patients with stroke (NIHSS < 5), TIA, or PVD. The intervention’s novelty lies in:

1. Integrated multi-component design: combining health checks, personalised coaching, psychosocial support, and practical service navigation within a single coherent framework.
2. Place-based delivery: home-based visits enabling environment assessment and addressing practical barriers inaccessible through clinic-based care, with telephone or NHS site flexibility alternatives available when preferred by participants.
3. Secondary care timing and delivery: delivered through hospital pathways during active engagement with vascular services. For stroke/TIA patients, intervention capitalises on the critical early secondary prevention window when recurrence risk and motivation are highest. For PVD patients, intervention provides support during ongoing disease management regardless of time since diagnosis.
4. Equality health advisors: dedicated public health professionals trained in health coaching, community asset navigation, and SDoH assessment, a distinct role from existing clinical teams.

This single-arm study was designed using the 33-item SPIRIT checklist [37] and the Medical Research Council’s framework for developing complex interventions [38].

Findings will directly inform decisions about progression to efficacy evaluation and guide optimisation of intervention design, recruitment strategies and delivery protocols (Table 1).

**Table 1:** SPIRIT: overview of Equality Health-CVD

	Study period					
	Enrolment	Intervention				End of study
Timepoint	T0	T1	T2	T3	T4	T5
Enrolment						
Eligibility screen	X					
Informed Consent	X					
Intervention						
Equality Health Home Visit Form		X	X	X	X	
Reflective diary		X	X	X	X	
Assessments: Case Report Form A (incl. AUDIT-C)₁ Health Check (Weight, HR, BP, BMI, Waist Circumference)^2^ EQ-5D₃ PROMIS-10₄ Social Wellbeing Scale₅ Case Report Form B (incl. AUDIT-C)₆	X X X X	X			X X X X X	
Semi-structured interview						
Participants ₇					X	
Equality Health Advisor₈						X

*SPIRIT*: overview of Equality Health-CVD. Subscript digit one (1) indicates the following: At baseline, socio-demographic information will be collected, including age, gender, ethnicity, living arrangements, pain level, smoking status and Audit C, Alcohol Use Disorders Identification Test.; superscript two (2) indicates the following: physical testing assessments to identify health risk factors and health status; subscript digit three (3) indicates European Quality of Life 5 Dimension Level 5; subscript digit four (4) indicates the following: PROMIS-10, Patient-Reported Outcomes Measurement Information System Global Health; subscript digit five (5) indicates the following: The South Wales Social Wellbeing Scale (SWSWBS); subscript t digit six (6) indicates the following: End of intervention evaluation items and Audit C, Alcohol Use Disorders Identification Test; subscript digit seven (7) indicates the following: participants semi structured interview questions that will be completed at the final follow up visit; subscript digit eight (8) indicates the following: Equality Health Advisor semi structured interview questions that will be completed at the end of the study

### Feasibility study aims

Before progressing to a full-scale effectiveness trial, this feasibility study will:

1. Assess whether the intervention can achieve adequate recruitment and retention in this population? (RQ1).
2. Determine whether the intervention can be delivered with fidelity and data collection is feasible (RQ2)
3. Examine intervention acceptability to participants using Theoretical Framework of Acceptability (RQ3)
4. Explore deliverers’ perspectives on implementation feasibility, identifying barriers, facilitators and refinement needs (RQ4).

### Study design

This feasibility study is a UK-based single-site pilot study of the Equality Health–CVD intervention. The intervention consists of four home visits encompassing health checks, coaching, support linked to social connections and wider determinants of health delivered by Equality Health Advisors over a 12-week period. Tables 2 and 3 logic model articulates the conceptual framework of the Equality Health–CVD intervention including how the context, intervention activities interlink with perceived impact and outcomes.

**Table 2:** Equality Health-CVD logic model

Context	Intervention	Mechanisms of impact	Intervention outcomes
Setting: Secondary care support service. Target population: People diagnosed with either of the following: • History of ischemic stroke NIHSS score of less than 5 • History of Transient ischemic attack • Peripheral Vascular Disease Number of Participants Total = 20 Aim: Improve Health and Wellbeing Age: Male and Female – Age 18 to 100 Deliverers: Equality Health Advisors Number of Visits: 4 visit in total – approximately 4 week apart Length of Visit: Approximately 1 hour	Link participants to community-based interventions Link participants to CIVIC interventions that support (Closeness, Identity, Valued relationships, Involvement, Cared for/accepted) – Civic duty Provide Health and Wellbeing coaching using basis CBT (Using NHS England health and wellbeing coaches framework) Physiological testing • Blood Pressure and HR • Height • Weight • BMI • Waist Circumference Act upon wider determinants of health Fuel poverty -Nest Income – Citizens Advice Poor housing and living conditions. Education Unemployment Health care service	Accessible pathways into service-based and community-based interventions Participative social functioning Knowing who to contact Participant is Listened to Supporting Participant motivation Flexible role Trusting interpersonal relationship Person-centred approach Adaptability of intervention Cross pathway referrals	Improved physical health Improved mental health and well-being Better Health Behaviours Increased social connectedness Reduce social isolation. Improved healthcare utilisation outcomes (such as fewer GP visits and lower emergency admission) Increase Self Efficacy in managing personal health Enhance goal achievement Reduction in risky health behaviours Improve health literacy Support health awareness through physiological testing Increased engagement in preventive health behaviours Improve disease management Greater awareness of social determinants of health Increase access to essential services (e.g., healthcare, transportation)

**Table 3:** Key components in the Equality Health Advisor Handbook

Key components
Quality standards
Health check guidance
Health coaching guidance
Psycho-social wellbeing guidance
Wider determinants of health guidance
Documentation and follow-up
Escalation process

## Recruitment and study setting

### Sociodemographic context of recruitment area

The study will recruit from hospital sites across the Aneurin Bevan University Health Board (ABUHB) catchment area, serving a population of approximately 565,412 people across five local authorities in Gwent, South Wales: Blaenau Gwent, Caerphilly, Monmouthshire, Newport, and Torfaen [39].

This area demonstrates substantial socioeconomic heterogeneity with significant pockets of deprivation. According to the Welsh Index of Multiple Deprivation (2025) [40]:

- Blaenau Gwent ranks as one of the most deprived local authorities in Wales, with the highest proportion of small areas in the most deprived 10% in Wales (20% representing 9 areas), followed by Newport (18% or 18 areas).
- Torfaen also shows significant deprivation, with (8.3%) of LSOAs falling within the most deprived 10% of LSOAs.
- Caerphilly demonstrates a mixed deprivation pattern, with former coalfield communities experiencing pockets of deep-rooted deprivation.
- Monmouthshire is relatively affluent but contains pockets of rural deprivation.

These deprivation patterns translate into substantial health inequalities. Data from the Gwent Joint Strategic Assessment 2024/25 [39] reveal:

- Female life expectancy in Gwent is 81.3 years, but healthy life expectancy is 57.9 years, meaning women could live up to 23.4 years in ill health.
- Torfaen shows the lowest healthy life expectancy for women at 53.3 years, 4.6 years below the Gwent average, indicating particularly pronounced health inequalities.
- Male life expectancy in Blaenau Gwent is 76 years (2 years below the Welsh national average); however, men spend an average of 19.3 years in ill health.
- Age standardised premature death rates from key non-communicable diseases that include stroke, TIA, PVD, cancer, diabetes and respiratory conditions between are 279.6 per 100,000 for women and 383.6 per 100,000 for men (2021–2023)

**Fig. 1:**
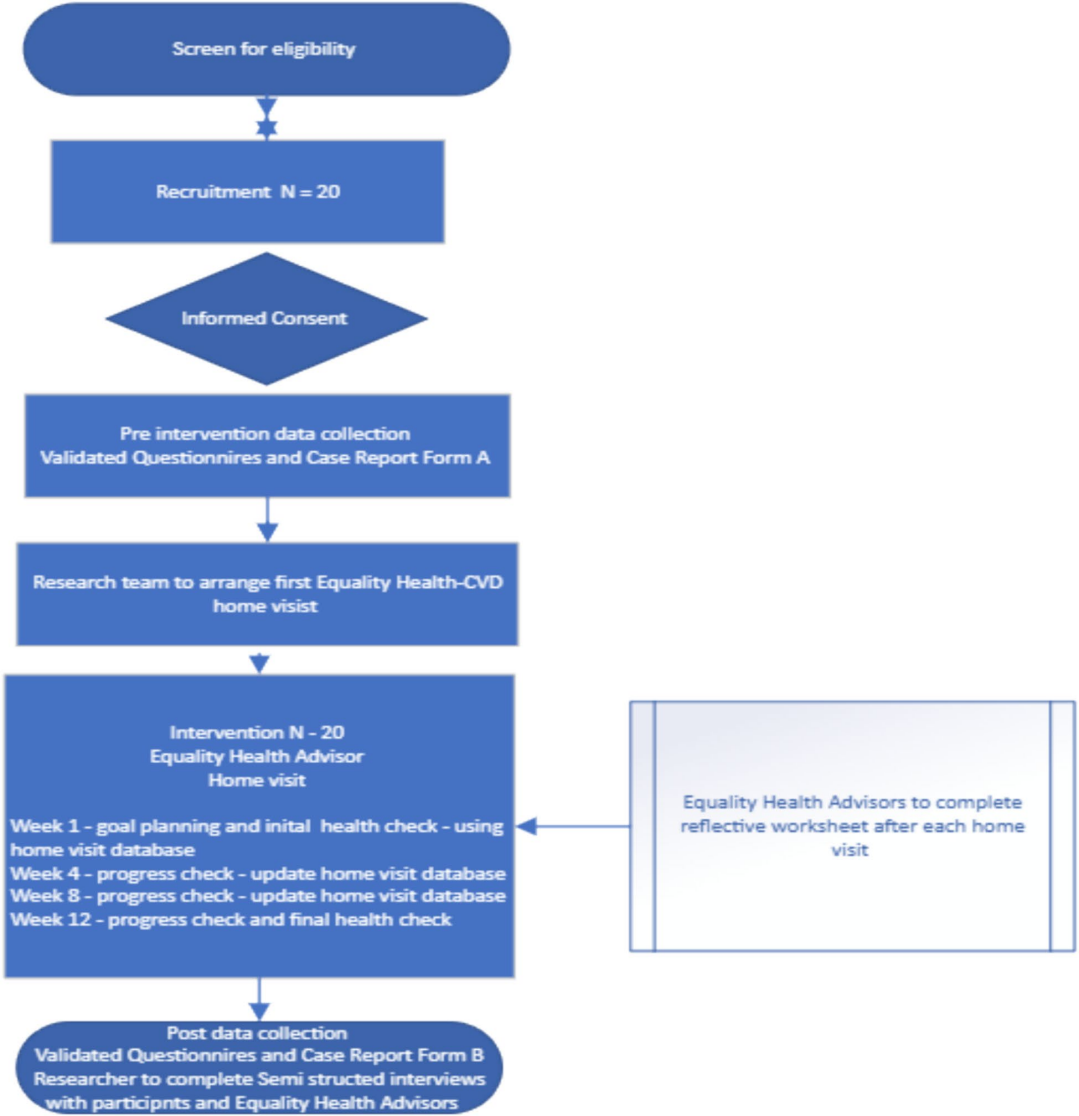
Participant timeline and study flow chart

The social diversity of the ABUHB catchment area provides opportunity to recruit participants experiencing significant SDoH challenges. Hospital and clinic-based recruitment through secondary care pathways provides access to patients from across this diverse socioeconomic spectrum (Fig. 1).

#### Participants

The study aims to recruit 20 participants between October 2024 and August 2025. Participants in the study will undergo eligibility screening in various hospital sites across the Aneurin Bevan University Health Board. Recruitment will be achieved by approaching all appropriate outpatient clinics. Eligible participants will be provided with verbal information about the study and given a copy of a patient information sheet by the Aneurin Bevan University Research Team. Participants will be given adequate time to review the information sheet and ask questions. Participants may choose to give consent on the same day or take the information home to consider. In this case, patients will have the option to be contacted by the research team or given the contact details of the research team to arrange a follow-up appointment. Consented participants will self-complete Case Report Form A, which includes demographic and SDoH profile data collection (housing, income, social support, service access barriers) and various validated social and wellbeing patient-reported outcome measures. Thereafter, participants will be offered the first Equality Health appointment at a convenient time for the participant.

Potentially eligible participants will reside in Gwent, where identification, screening, and consent will take place at various sites across Aneurin Bevan University Health Board in Wales during a routine outpatient appointment or as an inpatient.

#### Study population: inclusion and exclusion criteria

This feasibility study deliberately includes patients with three related vascular conditions: a history of ischaemic stroke, National Institutes of Health Stroke Scale (NIHSS) score of less than 5, a history of transient ischaemic attack (TIA), or a referral to a Peripheral Vascular Clinic. This heterogeneous approach is justified on theoretical and pragmatic grounds. All three conditions result from atherosclerotic vascular disease sharing common mechanisms and modifiable risk factors [9–12]. Additionally, the Equality Health-CVD intervention targets the person and their environment, not the specific vascular pathology. Heterogeneity will be mitigated through baseline characteristics reported by condition subgroup, recruitment/retention rates analysed by pathway and qualitative interviews will be examined for any emerging diagnosis-specific themes that may inform future trial design.

Participants eligible for recruitment in this study are those aged 18 years or older. Eligible participants must also have the capacity to provide informed consent and express a willingness to participate in a qualitative interview at the conclusion of the intervention. Exclusion criteria include patients receiving or eligible for palliative care. Unwillingness or inability (e.g. physical or cognitive) to comply with study procedures. Patients already participating in a clinical rehabilitation home intervention and with an unwillingness to take part in a qualitative interview at the end of the intervention are also excluded.

#### Sample size

The sample size of 20 participants is appropriate for a feasibility study and aligns with published guidance on feasibility trial design [41–43]. The participants will be selected through convenience sampling; this decision was made due to time and resource constraints [44]. The study will remain open for 12 months. Recruitment progress will be closely monitored, and strategies will be adapted as needed to ensure target enrolment is achieved. The study will remain open until October 2025, at which point it will close for data analysis.

#### Withdrawal of study participants

Participants may provide consent but later choose to withdraw from the study. The frequency and timing of the withdrawal will be recorded. If a participant chooses to withdraw from the study and not participate in further follow-ups, the data collected up to the point of withdrawal will still be retained.

#### Equality Health-CVD intervention

The intervention is delivered primarily through home visits, with alternative options for telephone or video call to ensure flexibility based on individual needs. Each session is tailored towards the specific needs of the participant, including a coaching session on what matters to them and goal setting. Health checks are conducted on week 1 and 12 to elicit a health-related conversation, and participants are provided with support linked to social connection and wider determinants of health. The Equality Health Advisor may assist participants in navigating different services which may include contacting service providers on their behalf.

The intervention comprises 4 sessions spaced approximately 4 weeks apart. Between sessions participants work on their identified goals and engage with public or community services. The follow-up sessions will provide additional support, monitor progress, and address any emerging needs and barriers. Should barriers arise, the participant and the Equality Health Advisor will collaborate to identify solutions. Information about delivery time, mode, application of intervention components, and retention/adherence rate will all be gathered by the Equality Health Advisor using the equality health home visit form. These records will later be transferred to an excel sheet. At the end of each session the Equality Health Advisors complete a reflective log.

At the final session (week 12) participants will reflect and discuss their achievements, any potential setbacks encountered, and strategies to maintain or build upon their achievements and progress. The participant will also undertake a second health check and complete Case Report Form B with the Equality Health Advisor.

As part of the final intervention process evaluation, a subset of participants (approximately 10–15) will be selected randomly using a non-probability convenience sample [44] and will participate in a semi-structured interview during the 12-week follow-up visit. The semi-structured interviews will be conducted by the lead researcher and will either be conducted face to face in the participants’ home using an audio device or via video link.

The interviews will explore the following themes:

- Participant’s experiences and perceptions of the Equality Health-CVD Intervention
- Acceptability of the intervention and study design.
- Challenges or opportunities of future research in reducing health inequalities in the community.

In addition, the Equality Health Advisors will also participate in semi-structured interviews to explore their views of the Equality Health-CVD study using a similar thematic qualitative approach.

During the development of the protocol, the lead researcher conducted a public and community asset mapping exercise to identify services, activities, and local contacts available across the five local authorities in Gwent. All information was added to templates with the aim to help the Equality Health Advisors to navigate public and community asset provision.

A series of patient and professional consultations were organised to inform the co-design of the protocol and intervention. To support the intervention, health check equipment was purchased, and an Equality Health Adviser Handbook was created. This handbook aims to provide a framework for delivering the Equality Health-CVD intervention focusing on four dimensions of the adapted Labonte model (Table 4).

**Table 4:** Intervention procedures

Procedure	Timepoints (Weeks)
Eligibility criteria review	Week 0
Informed consent	Week 0
Medical history	Week 0
Case Report Form A (incl. AUDIT-C)	Week 0
Health Check (Weight, HR, BP, BMI, Waist Circumference)	Weeks 1 and 12
EQ-5D	Weeks 0 and 12
PROMIS-10	Weeks 0 and 12
South Wales Social Wellbeing Scale	Weeks 0 and 12
Equality Health Home Visit	Weeks 1, 4, 8, 12
Case Report Form B (incl. AUDIT-C)	Week 12
Semi-structured interview	Week 12

The Equality Health Advisors are employed by the Aneurin Bevan University Health Board within the Public Health Team. Prior to study implementation, the Equality Health Advisors received additional training to support safe and effective communication with participants, which included a health coaching course, first aid emergency responder, and various online courses in clinical skills, service navigation, and community engagement.

To balance protocol fidelity with real-world feasibility, consultations may be scheduled within a ±14-day window around target dates.

- Consultation 2: target week 4 (deliverable weeks 2–6)
- Consultation 3: target week 8 (deliverable weeks 6–10)
- Consultation 4: target week 12 (deliverable weeks 10–14)

This flexibility reduces unnecessary barriers to participation, including work schedules, caregiving responsibilities, health fluctuations, or any additional challenges that may arise (Table 4).

### Outcomes

#### Primary

The primary outcome is to evaluate the feasibility and acceptability of the Equality Health–CVD study in terms of recruitment and retention rate to the intervention (Study objective 1). A record will be kept of the number of participants recruited within 9 months of the study opening, including the number of participants who participated in the 12-week follow-up. Additionally, a feasibility assessment of intervention fidelity will be conducted (Study objective 2). This will involve documenting the number of home visits completed, recording the data collected during each visit, and tracking the outcomes of signposting to community and public assets.

Secondary outcomes: the secondary outcomes will examine the experiences and acceptability of the intervention from the perspectives of both participants and deliverers (Equality Health Advisors). This evaluation linked to (objective 3 and 4) will include identifying barriers and facilitators to delivery as well as perceived impacts. Data will be collected through qualitative semi-structured interviews conducted at the end of the intervention for participants and at the end of the study for Equality Health Advisors. To complement these findings, reflective diaries maintained by Equality Health Advisors during home visits will also be analysed.

In addition, participants will complete a series of validated questionnaires at baseline and 12 weeks post-baseline as part of the feasibility study's evaluation. These quantitative Patient-Reported Outcome Measures (PROMs) will be used in conjunction with qualitative semi-structured interviews as data descriptors. This will facilitate whether the participants are willing and able to complete these measures, in addition to capturing subjective health and social wellbeing outcomes that may enhance data richness.

#### Progression criteria

To recognise and acknowledge protocol success, the following criteria will be applied using a green light system.

**Table:** 

Linked RQ	Progression criteria		Green light	Amber light	Red light	Data
	N	Detail				
1. Recruitment	1	% of target number of participants recruited (target: 20; 9 -month recruitment period)	>75%	60–75%	< 60%	*Screening log* *Baseline, RN – Informed Consent Form CRF A*
1. Retention	2	% of patients who participate in 12-week follow-up (of those who complete min. 1 intervention session)	>65%	50–65%	<50%	12-week follow up questionnaire
2. Feasibility of intervention implementation	3	The intervention is implemented with fidelity (i.e., 4 home visits conducted with data collected recorded at each visit and signposting to community and public assets)	No traffic light criteria to be applied			HEA reflective diaries 12-week interview data
3. Acceptability of the intervention	4	Data indicates the intervention is acceptable to participants and deliverers				HEA reflective diaries 12 week interview data

The Green light system (progression criteria) indicators are as follows:

- Green—indicates current procedure can be used for future study
- Amber—indicates current procedure needs attention with discussion for optimisation in future study
- Red—indicates current procedure is unacceptable and not feasible therefore an alternative will need to be tested within a future study

The Study Management Group (SMG), which includes an ABUHB Quality Assurance Officer, an Independent Data Validation Reviewer (Quantitative), and an Independent Data Validation Reviewer (Qualitative), will review all aspects of the progression criteria.

## Research methods

### Quantitative data

The following questionnaires will be completed at baseline and post-baseline week 12.

- Case Report Form A will collect demographic data including wider determinants of health.
- PROMIS 10—the PROMIS 10 is a comprehensive and accessible set of tools used to measure self-reported physical, mental, and social health, including symptoms, function, and general perceptions of health and wellbeing [45].
- South Wales Social Wellbeing Scale—The South Wales Social Wellbeing Scale (SWSWBS) is a tool to the quality of respondents’ overall experience of social wellbeing via the external social resources they possess, their perceived ability to engage in and enjoy the social world in which they live, and, as a result, their capacity for human functioning and flourishing [46].
- EuroQol-5D—EQ-5D is an instrument which evaluates the generic quality of life developed in Europe and widely used. The EQ-5D descriptive system is a preference-based HRQL measure with one question for each of the five dimensions that include mobility, self-care, usual activities, pain/discomfort, and anxiety/depression [47].
- Case Report Form B will collect data satisfaction of feasibility and implementation.

### Physical testing

As part of the intervention, the Equality Health Advisor will conduct a series of tests. The purpose of the physical tests is to identify participants at risk, set goals, elicit health improvement conversations, monitor progress, and establish baseline measures for future testing as part of the intervention (Table 5).

**Table 5:** Physiological testing

Physiological testing
Blood pressure
Heart rate
Height
Weight
Body mass index
Waist circumference

CE-marked/UKCA calibrated devices and equipment will be used. The Equality Health Advisor will consider the testing environment and physical limitations of participants prior to conducting a test. All tests aim to be safe, reproducible, validated, reliable, sensitive, and practical to undertake.

### Qualitative data collection

#### Participant interviews

Semi-structured interviews will be conducted using a sub-sample of participants (*n* = 10–15) and will examine intervention acceptability using the Theoretical Framework of Acceptability [48]. The TFA provides a systematic approach to understanding intervention engagement using seven constructs. Interview questions aim to allow participants to describe experiences naturally. TFA coding will be applied retrospectively during analysis, an approach endorsed by Sekhon et al. [48].

In addition to acceptability assessment, participants' interviews will examine whether the intervention operated according to its theoretical framework, (the adapted Labonte Model) (RQ2). The analysis will assess which dimensions participants experienced most strongly, whether all four were activated in practice, and how dimensions interacted.

#### Equality health advisor interviews

Semi-structured interviews with Equality Health Advisors will examine implementation feasibility from deliverers’ perspectives, using inductive thematic analysis [49, 50]. In addition, Equality Health Advisor reflection diaries will be analysed to confirm intervention delivery intention, including challenges, successes, and suggestions for improvement.

Interviews will be conducted in person, though a video/telephone call option will also be available. Interviews will be audio recorded and transcribed, and information will remain confidential. Analysis will employ a combined deductive-inductive approach:

- Deductive Frameworks: TFA seven constructs and Labonte four dimensions (participant interviews)
- Inductive themes: emergent theme beyond frameworks (participant interviews) and implementation insights from Equality Health Advisor data.

Coding will involve systematic assignment of data segments to framework constructs and/or emergent themes, with patterns identified across participants. Reporting will follow the guidelines set by the Consolidated Criteria for Reporting Qualitative Research (COREQ) [51]. Member checks will also be conducted to ensure credibility and confirmability. Data collection will continue until saturation is reached. If new data continue to emerge after the final interview, additional interviews may be conducted. Triangulation across quantitative feasibility metrics, participant TFA findings and Equality Health Advisor implementation insights aims to provide a thorough summary of intervention acceptability and deliverability, informing progression decisions and refinement recommendations.

## Discussion

Research suggests that place-based interventions can positively impact physical and mental health while also addressing the broader determinants of health outcomes [29–32]. This feasibility study addresses a specific evidence gap: no existing secondary care intervention operationalises all four Labonte model dimensions for patients with vascular conditions. The findings will determine whether the Equality Health-CVD intervention is sufficiently feasible and acceptable to justify progression to an effectiveness trial.

The study will use mixed methods to assess feasibility outcomes for vascular patients and healthcare commissioners. Quantitative measures will establish recruitment, retention, and fidelity metrics against pre-specified progression criteria, while qualitative interviews will assess acceptability through the Theoretical Framework of Acceptability and examine theoretical fidelity to the adapted Labonte model. Together, these data streams will provide early evidence to inform intervention optimisation and future trial design.

### Study limitations

Hospital and clinic-based recruitment may introduce selection bias, potentially under-representing individuals experiencing the most significant social determinants of health challenges. Baseline demographic and SDoH data will characterise the recruited sample and inform future recruitment strategies. The single-arm design and small sample size (*n* = 20), while appropriate for feasibility assessment, preclude conclusions about intervention effectiveness or generalisability beyond the study population. Geographic restriction to one health board limits transferability to other settings. The inclusion of three vascular diagnoses (stroke, TIA, PVD) introduces clinical heterogeneity, with subgroup numbers insufficient for meaningful diagnosis-specific analysis. Baseline characteristics and outcomes will be reported descriptively by condition, with qualitative interviews examined for emerging diagnosis-specific themes; if clear differences between stroke/TIA and PVD populations are identified, pathway-specific adaptation will be recommended for future trials.

### Ethics declarations and confidentiality

The study was approved by the East of Scotland Research Ethics Committee 1 (11TH September 2024) Registration number 24/SS/0067. The trial was registered with the ISRCTN registry (15th November 2024) with trial identification number ISRCTN11889657.

The research team will follow GDPR 2018 and Good Clinical Practice requirements. Data will be stored on a secure NHS server, accessible only to the research team for the duration of the study. Participants will be provided with a unique ID to protect confidentiality before, during and after participation. Data will be collected using an ABUHB laptop using Microsoft Word, Excel, and audio software. Data collected will be kept strictly confidential. Personal data will be retained only for the minimum time necessary.

### Data management and monitoring

The study team will adhere to the Equality Health CVD study's data management and monitoring plan. There are two data management levels in this study:

- Level 1: holds identifiable patient data, including screening, consent, demographic information, and PROMs collected from CRF A and B. In addition, to the end of study audio recordings and transcripts of the semi-structured interview from participants and Equality Health Advisors.
- Level 2: collects data from Equality Home Visits. No identifiable patient data is stored here; participants are referenced by a participant number. Information will be populated by the Equality Health Advisors.

The Study Management Group will oversee amendments, data quality, meeting monthly to assist the Study Project Team with data queries, escalations, and adverse event support. Amendments requiring NHS REC review will only be implemented once full REC approval is obtained. A risk assessment plan will remain active throughout the study to identify any potential threats to the organisation or the health and safety of researchers and participants.

## Data Availability

Please contact Anna Pennington for Equality Health-CVD intervention details. The final dataset will remain the property of Cardiff University.
